# Generation and Characterization of UL21-Null Herpes Simplex Virus Type 1

**DOI:** 10.3389/fmicb.2012.00394

**Published:** 2012-11-16

**Authors:** Yoshifumi Muto, Fumi Goshima, Yoko Ushijima, Hiroshi Kimura, Yukihiro Nishiyama

**Affiliations:** ^1^Department of Virology, Nagoya University Graduate School of MedicineNagoya, Japan

**Keywords:** HSV-1, UL21, intermediate filaments, glial cells, GFAP

## Abstract

UL21 of herpes simplex virus type 1 (HSV-1) is an accessory gene that encodes a component of the tegument. Homologs of this protein have been identified in the alpha, beta, and gamma herpesvirus subfamilies, although their functions are unclear. To clarify the functions of UL21, we generated a UL21-null HSV-1 mutant. Growth analysis showed that the synthesis of infectious UL21-null HSV-1 in glial cells was delayed and that the overall yield was low. The plaque sizes of the UL21-null mutant were smaller than those of wild-type HSV-1. We identified several candidate UL21-interacting proteins, including intermediate filaments, by yeast two-hybrid screening. The distribution of glial fibrillary acidic protein (GFAP), which is the main component of intermediate filaments, was altered in UL21-null mutant-infected glial cells compared to wild-type virus-infected cells. These results will help clarify the function of UL21 and broaden our understanding of the life cycle of HSV.

## Introduction

Herpes simplex virus type 1 (HSV-1) is an enveloped, large DNA virus with a genome consisting of approximately 80 genes that encode transcriptional regulatory proteins, capsid proteins, enveloped glycoproteins, viral DNA replication proteins, and proteins involved in the cleavage/packaging of viral DNA. However, less than half of these genes are essential for replication of the virus in culture (Roizman et al., 2006). The remainder are referred to as accessory genes, which are dispensable for replication in cultured cells but necessary for replication and spread in the host (Mori and Nishiyama, 2006).

UL21 of HSV-1 is an accessory gene that encodes a 535-amino acid component of the tegument (Baines et al., 1994). Homologs of this protein have been identified in the alpha, beta, and gamma herpesvirus subfamilies (Baer et al., 1984; Davison and Scott, 1986; Chee et al., 1990). Most work on UL21 has been performed using HSV-1 and pseudorabies virus (de Wind et al., 1992; Wagenaar et al., 2001; Harper et al., 2010; Mbong et al., 2012). Absence of UL21 results in a delay early in the HSV-1 replication (Mbong et al., 2012), while processing of newly replicated viral DNA is impaired in UL21-null pseudorabies virus (de Wind et al., 1992). However, the role of this protein is still unclear.

Previously, we found that UL21 has sequence similarities with tau (Takakuwa et al., 2001), a family of neuronal proteins that associate with microtubules and enhance microtubule formation and stability *in vitro* (Cleveland et al., 1977; Butner and Kirschner, 1991). UL21 promotes the outgrowth of long cellular processes and associates physically with microtubules. In this way, UL21 may facilitate intracellular transport of the virus (Takakuwa et al., 2001).

In this study, to clarify the functions of UL21, we generated a UL21-null mutant and characterized its properties. Furthermore, we screened for UL21-interacting host proteins using a yeast two-hybrid system. We also compared the gene product distributions in UL21-null mutant-infected and wild-type HSV-1-infected cells.

## Materials and Methods

### Cells and viruses

Vero (African green monkey kidney) and A172 (human glioblastoma) cells were obtained from the RIKEN BioResource Center (Ibaraki, Japan). Vero cells were maintained in Dulbecco’s modified Eagle’s minimum essential medium supplemented with 5% calf serum, 100 U/ml penicillin, 100 μg/ml streptomycin, and 2 mM glutamine at 37°C in 5% CO_2_. A172 cells were maintained in RPMI1640 supplemented with 10% fetal calf serum at 37°C in 5% CO_2_. Wild-type HSV-1 strain 17syn^+^ was kindly provided by C. Cunningham. The virus stocks were propagated and titrated on Vero cell monolayers.

### Antibodies

Anti-UL21 polyclonal rabbit antibodies were generated as described previously (Takakuwa et al., 2001). The following polyclonal antibodies were used in this study: rabbit anti-glial fibrillary acidic protein (GFAP; Cell Signaling Technology, Inc., Danvers, MA, USA), rabbit anti-G protein-coupled receptor 56 (GPR56; Medical and Biological Laboratories, Nagoya, Japan), rabbit anti-neurofilament light polypeptide (NEFL; Cell Signaling Technology, Inc.), Alexa Fluor^®^ 555-conjugated goat anti-mouse IgG1 (Life Technologies, Grand Island, NY, USA), and Alexa Fluor^®^ 555-conjugated goat anti-rabbit IgG (Life Technologies). Monoclonal mouse antibodies against the following proteins were used: VP5, β-actin (Abcam, Cambridge, UK), α-actinin-1 (Upstate, Temecula, CA, USA), vimentin (Sigma, St. Louis, MO, USA), and β-actin (Sigma). Normal goat serum was obtained from DAKO (Glostrup, Denmark).

### Construction of the UL21-null mutant

Two HSV-1 strains, UL21D and UL21R, were constructed. Viral DNA from HSV-1 strain 17 was purified for use as the template. The 1.0-kb long 5′ end of UL21, including the promoter region, was amplified by PCR using a forward primer (5′-ccggaattcggctaagatccaccccaac-3′) carrying an *Eco*RI site and a reverse primer (5′-cggggtacccgcgggcggcgacgtaacac-3′) carrying a *Kpn*I site. The amplified product was digested with *Eco*RI and *Kpn*I, and cloned into pEGFP-N3 (Invitrogen). Next, the 1.0-kb long 3′ end of UL21, including the non-coding region, was amplified by PCR using a forward primer (5′-aaggaaaaaagcggccgcaagaccccaataaacg-3′) carrying a *Not*I site and a reverse primer (5′-caacttaagctgcctctccgacctgc-3′) carrying an *Afl*II site. The amplified product was digested with *Not*I and *Afl*II and cloned into pEGFP-N3. The resultant plasmid, pEGFP-non-coding-UL21, contained the EGFP gene flanking the 5′ and 3′ ends of the non-coding region of UL21. The purified plasmid DNA was linearized and cotransfected by the DEAE-dextran method (Kawaguchi et al., 2001) onto rabbit skin cells with the purified genome of HSV-1 strain 17. EGFP-positive plaques were purified by three rounds of plaque purification; the stocks (100% purity) were designated UL21D. In the mutant virus, the open reading frame of UL21 was replaced with the coding region of EGFP. For the construction of a revertant virus, the purified genome of UL21D was cotransfected with a 2.3-kbp fragment containing the wild-type HSV-1 UL21 gene onto rabbit skin cells. Three rounds of plaque purification were performed to obtain reverted virus stocks, which were designated UL21R.

### Extraction of cell lysates and Western blot analysis

For Western blotting, cells were lysed with SDS sample buffer containing 50 mM Tris-HCl, pH 6.8, 2% SDS, 10% glycerol, 6% 2-mercaptoethanol, and 0.0025% bromophenol blue. The lysates were separated by SDS-PAGE and transferred to polyvinylidene difluoride membranes (Immobilon-P membranes; Millipore, MA, USA). The membranes were blocked with blocking buffer (5% skim milk and 0.1% Tween 20 in PBS) for 1 h at room temperature. After incubation with appropriate primary antibodies for 1 h at room temperature, the membranes were incubated with horseradish peroxidase-conjugated secondary antibodies, which were subsequently detected using West-one (iNtRON Biotechnology, Inc., Sungnam, Korea). The membrane was stripped with Restore PLUS Western Blot Stripping Buffer (Thermo Fisher Scientific Inc., Waltham, MA, USA), reblocked, and reprobed with different primary antibodies.

### One-step and multistep growth assays

Analyses of the one-step and multistep growth kinetics were performed as described (Nozawa et al., 2005). Vero or A172 cells were infected with each virus at multiplicities of infection (MOI) of 3 or 0.03 and incubated for 1 h at 37°C to allow virus adsorption. The culture medium was then replaced with newly prepared medium containing 2% calf serum. The cells and supernatants were harvested at the indicated times after infection. The viral progeny were titrated on Vero cells by plaque assays. Each experiment was performed three times, and a representative result was shown.

### Yeast two-hybrid screen

A ProQuest Two-Hybrid system with Gateway technology (Life Technologies) was used (Parr et al., 2006; Wang and Chory, 2006). The DNA fragment encoding amino acids 1-535 of UL21 was subcloned in-frame into the pDBLeu vector and used as a bait. A human fetal brain cDNA library was constructed with pPC86 and used as a prey vector. The yeast strain *MaV203* were co-transformed with the bait and prey vectors. Interactions were tested on SD medium minus Leu, Trp, and His, and containing 40 mM 3-Amino-1,2,4-Triazole, according to the manufacturer’s manual. Positive clones were selected by β–galactosidase activity. The library plasmids from these colonies were rescued, amplified by PCR, and sequenced (Ushijima et al., 2008).

### Immunofluorescence microscopy

Cells grown on coverslips were washed in PBS three times and fixed for 10 min in 4% paraformaldehyde in PBS at room temperature. For indirect immunofluorescence microscopy, the fixed cells were permeabilized in 1% Triton X-100 in PBS for 5 min at room temperature. The coverslips were inverted and touched to droplets (20 μl) of blocking buffer (4% goat serum and 1% bovine serum albumin in PBS) on a clean Parafilm sheet for 45 min at room temperature. Primary and Alexa Fluor^®^-conjugated secondary antibodies were diluted in blocking buffer and reacted for 60 min at room temperature. The samples were examined under a Zeiss LSM 510 confocal immunofluorescence microscope (Yamauchi et al., 2008).

## Results

### Construction of the UL21-null mutant and its confirmation

**Figure 1:**
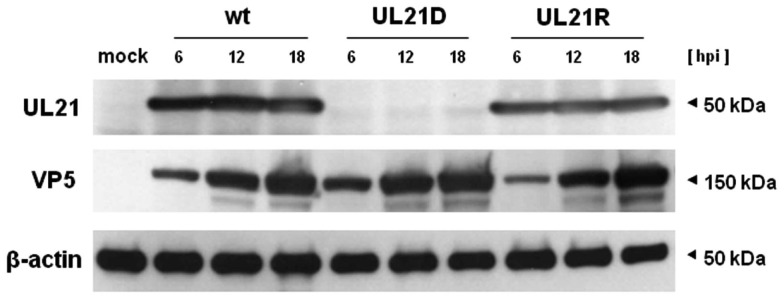
**Expression of UL21 and VP5**. Vero cells were infected with wild-type HSV-1 (wt), UL21D, or UL21R at an MOI of 3. The cells were harvested at 6, 12, and 18 h post-infection and subjected to SDS-PAGE and Western blotting, followed by detection with polyclonal rabbit or monoclonal mouse antibodies.

We constructed a UL21-null mutant virus by homologous recombination with an EGFP cassette flanking the 5′ and 3′ non-coding regions of UL21 (designated UL21D). We also constructed a reverted virus (UL21R). Viral DNA from UL21D and UL21R were used to amplify the manipulated area by PCR; sequencing of the products showed that the desired genetic manipulations had been made. To confirm the deletion and reversion, Western blot analyses were performed. The product of UL21 was not expressed in UL21D-infected cells, but it was detected in both wild-type- and UL21R-infected cells (Figure 1). VP5, a major capsid protein, was detected in UL21D-, wild-type-, and UL21R-infected cells (Figure 1).

### Growth kinetics of HSV-1 UL21

**Figure 2:**
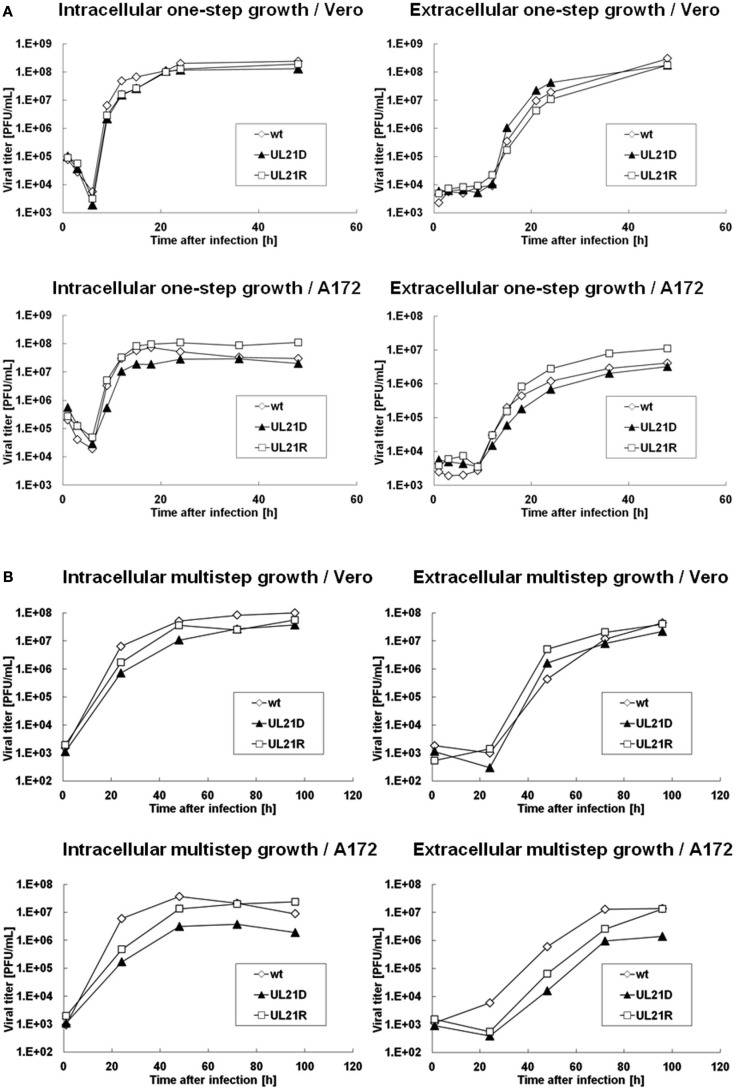
**Growth analyses of wild-type HSV-1, UL21D, and UL21R**. **(A)** One-step growth analysis. **(B)** Multistep growth analysis. Vero or A172 cells were infected with wild-type HSV-1 (wt), UL21D, or UL21R at MOI of 3 **(A)** or 0.03 **(B)**. At the indicated times post-infection, the infected cells and supernatant were analyzed separately by plaque assay to determine the intracellular and extracellular viral yields, respectively.

To determine whether the UL21-null mutant possessed a replication defect, we analyzed the growth kinetics of UL21D using Vero and A172 cells. For one-step growth analysis, cells were infected with wild-type HSV-1, UL21D, or UL21R at a high MOI. In A172 cells, the intracellular and extracellular yields of UL21D were reduced at 9–18 h post-infection and at 12–18 h post-infection, respectively (Figure 2A). In contrast, no such reduction was apparent in Vero cells (Figure 2A). For multistep growth analysis, cells were infected with virus at a low MOI. Although the growth kinetics of UL21D were similar to those of wild-type HSV-1 and UL21R in Vero cells, both the intracellular and extracellular viral titers of UL21D were lower in A172 cells (Figure 2B).

**Figure 3:**
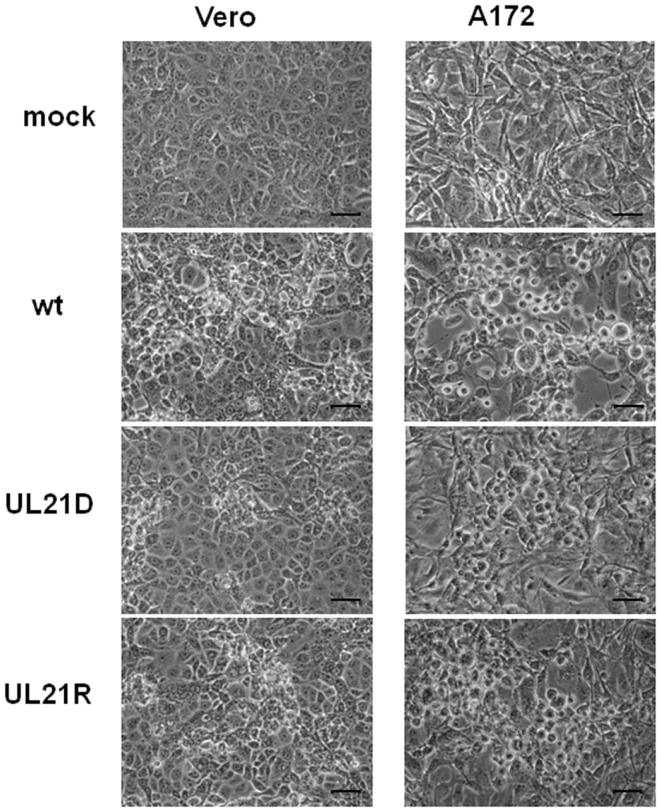
**Plaque formation by UL21D in Vero and A172 cells**. Vero or A172 cells were infected with wild-type HSV-1 (wt), UL21D, or UL21R at an MOI of 0.03. Viral plaques were photographed at 24 h post-infection. Bars indicate 50 μm.

Next, we compared plaque morphologies. When Vero or A172 cells were infected with UL21D at a low MOI, the plaques were smaller compared to both wild-type HSV-1 and UL21R (Figure 3).

### Screening for UL21-interacting proteins

A yeast two-hybrid screen of a human brain cDNA library using UL21 as bait identified eight candidate UL21-interacting proteins: α-1-actinin, GFAP, GPR56, MAX-like protein X, myelin basic protein, myo-inositol 1-phosphate synthase A1, NEFL, signal-induced proliferation-associated 1 like 2, and vimentin.

We chose to further examine the cytoskeletal proteins α-1-actinin, GFAP, NEFL, and vimentin because we previously showed that UL21 protein interacts with microtubules (Takakuwa et al., 2001). Additionally, GPR56 was chosen because this protein was reported to colocalize with α-1-actinin in glioma cells (Shashidhar et al., 2005).

### UL21 deletion altered the distribution of intermediate filaments in infected A172 cells

**Figure 4:**
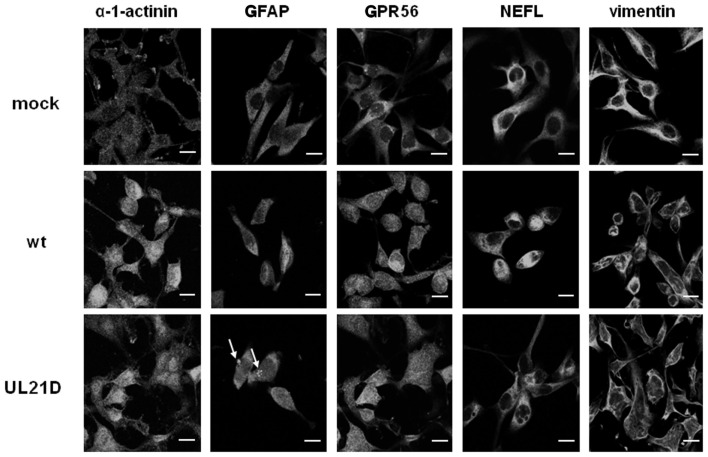
**Distribution of candidate proteins interacting with UL21 in glial cells**. A172 cells were infected with wild-type HSV-1 (wt) or UL21D at an MOI of 3. The cells were fixed at 12 or 18 h post-infection, reacted with anti-α-1-actinin, -GFAP, -GPR56, -NEFL, or -vimentin antibodies followed by Alexa Fluor^®^-conjugated secondary antibodies, and examined under a confocal immunofluorescence microscope. Arrows indicate GFAP accumulation. Bars indicate 20 μm.

Lastly, we investigated the distribution of α-1-actinin, GFAP, GPR56, NEFL, and vimentin in UL21D- or wild-type HSV-1-infected A172 cells (Figure 4). In UL21D-infected A172 cells, GFAP, a major intermediate filament in glial cells, accumulated in the cytoplasm. This accumulation was not seen in wild-type virus-infected cells. The distribution pattern of vimentin, another intermediate filament protein, differed slightly between UL21D- and wild-type virus-infected cells, although this could have been caused by a delay in the cytopathic effects of the UL21-null mutant.

## Discussion

Baines et al. (1994) who constructed the first UL21-null HSV-1 mutant, found that UL21 was dispensable for replication in cultured cells. They showed that the one-step growth of UL21-null HSV-1 in Vero cells was similar to that of the wild-type, but that HEL cells infected with the null mutant yielded approximately three- to five-fold less virus than wild-type or revertant virus-infected cells. Recently, Mbong et al. (2012) generated UL21-null virus using a BAC system and showed that the yields of their mutant virus in Vero cells were reduced as early as 6 h post-infection compared to wild-type and UL21 repair viruses, indicating that the absence of UL21 causes a delay in the production of infectious virus. In this study, the yields of UL21D were low at 9–18 h post-infection in glial cells, compared to the wild-type and revertant viruses. Growth kinetics of UL21D at low MOI were almost similar to those of wild-type HSV-1 and UL21R in Vero cells. However, at 24 h post-infection, the yield of UL21D was low, although final yields reached the level of wild-type HSV-1 and UL21R (Figure 2B). The delay in production of infectious virus accounts for the small plaque size seen in our study.

By yeast two-hybrid screening, we identified several proteins that could interact with UL21. It is notable that some were cytoskeletal proteins expressed in the central nervous system. GFAP is the major intermediate filament of mature astrocytes, and its relatively specific expression in these cells suggests an important function in the central nervous system. However, its exact function remains poorly understood, although GFAP may determine the complex morphology of astrocytes (Messing and Brenner, 2003). The influence of HSV infection on GFAP expression differs among models. HSV infection results in upregulation of GFAP synthesis in murine astrocytes (Carlucci et al., 1999), while its expression is reduced by HSV in rabbit retinal astrocytes (Wakakura et al., 1987). In contrast, HSV infection does not alter the general organization of GFAP in astrocytes (McCarthy et al., 1990). In this study, the results of our yeast two-hybrid screen indicated the association of UL21 with GFAP. Furthermore, the distribution of GFAP was altered in UL21-null mutant-infected A172 cells. These results suggest that UL21 is associated with intermediate filaments, including GFAP, and that HSV infection influences the organization of these proteins. The UL21 gene product is a tegument protein that lines the space between the envelope and nucleocapsid (Baines et al., 1994) and is associated with capsid proteins (Harper et al., 2010). UL21 protein binds to microtubules and promotes the outgrowth of long cellular processes (Takakuwa et al., 2001; Roizman et al., 2006). The findings of these studies and our results suggest that the function of UL21 may be associated with nucleocapsid transport by means of its interaction with cytoskeletal proteins.

In conclusion, we generated UL21-null HSV-1 and characterized its properties. The synthesis of infectious UL21-null HSV-1 was delayed and its overall yields were low, in glial cells. Yeast two-hybrid analysis identified several candidate UL21-interacting proteins, including intermediate filaments. The distribution of intermediate filaments was altered in UL21-null mutant-infected glial cells compared to wild-type virus-infected cells. Although further study is necessary, these results will help clarify the function of UL21 and enhance our understanding of the HSV life cycle.

## Conflict of Interest Statement

The authors declare that the research was conducted in the absence of any commercial or financial relationships that could be construed as a potential conflict of interest.
